# Complete Genome Sequence of *Streptomyces ambofaciens* DSM 40697, a Paradigm for Genome Plasticity Studies

**DOI:** 10.1128/genomeA.00470-16

**Published:** 2016-06-02

**Authors:** Annabelle Thibessard, Pierre Leblond

**Affiliations:** a Université de Lorraine, Dynamique des Génomes et Adaptation Microbienne, Unité Mixte de Recherche, Vandœuvre-lès-Nancy, France; b Institut National de la Recherche Agronomique, Dynamique des Génomes et Adaptation Microbienne, UMR 1128, Vandœuvre-lès-Nancy, France

## Abstract

The sequence of *Streptomyces ambofaciens* DSM 40697 was completely determined. The genome consists of an 8.1-Mbp linear chromosome with terminal inverted repeats of 210 kb. Genomic islands were identified, one of which corresponds to a new putative integrative and conjugative element (ICE) called pSAM3.

## GENOME ANNOUNCEMENT

We report here the complete genome sequence of *Streptomyces ambofaciens* DSM 40697 (1), which is used as a model for genome plasticity and genome evolution studies. A total of 84 contigs for 7.98 Mb with an average genome coverage of about 100× was obtained by assembling reads from Illumina Genome Analyzer sequencing of libraries of small (300-bp) and large (8-kb) DNA fragments. Sequence assembly and contig ordering were achieved using the CLC Main Workbench (Qiagen). A whole-genome map was constructed using the OpGen Argus system (Opgen, USA). Finally, gaps were closed by PCR and Sanger sequencing (Beckman Coulter). Coding sequence prediction and annotation were automatically performed using NCBI Prokaryotic Genome Annotation Pipeline (http://www.ncbi.nlm.nih.gov/genome/annotation_prok/).

The *S. ambofaciens* DSM 40697 genome consists of a single linear replicon of 8,137,876 bp, with long terminal inverted repeats of 212,655 bp (2). Six rRNA operons, 65 tRNAs, and 6,911 protein-coding genes were found on the chromosome. This strain shows common genomic traits with its related strain *S. ambofaciens* ATCC 23877 (3). Hence, the two strains possess a linear chromosome of slightly more 8 Mb (8.1 Mb and 8.3 Mb for strains DSM 40697 and ATCC 23877, respectively) and with the same G+C content (72%). Some relevant genetic traits, however, differ: the DSM 40697 strain is devoid of the pSAM1 plasmid (89 kb), the integrative and conjugative element pSAM2 (ICE, 11 kb), and the CIs-mobilizable element (CIME) called XSAM1 (42 kb [4]), immediately adjacent to pSAM2. In contrast, *S. ambofaciens* DSM 40697 possesses specific regions, some of which exhibit compositional and structural characteristics shared with genomic islands (e.g., low G+C content, flanking direct repeats, and frequently transferred genes). Such a region showed most of the typical elements of actinomycete integrative and conjugative elements (AICEs [5]) and was tentatively named pSAM3. This element is 18,965 bp long and is inserted in a tRNA-Arg gene (SAM40697_tRNA54, from positions 5244106 to 5244180). The pSAM3 element includes functional homologues to most of the key actors involved in AICE biology: integration (*int*), excision (*xis*), replication (*repSA*), transfer (*traS*), intramycelial transfer (*spdABCD*), and regulation (*pra*). The element presents an average G+C content of 65.25%. It was shown to excise between flanking direct repeats (21 bp) and to circularize when *S. ambofaciens* DSM 40697 was cocultured with strains devoid of pSAM3 (i.e., *S. ambofaciens* ATCC 23877 or *S. lividans* TK23).

The availability of the genome sequence of *S. ambofaciens* DSM 40697 allowed us to map loci involved in early reports of genomic plasticity (reference 6 and references therein). Hence, the two loci involved in intense DNA amplifications, AUD6 and AUD90, as well as recombination points (homologous and illegitimate) associated with chromosomal arm replacement (*hasR* and *hasL* [7]) and fusion (NSA27 fusion point [8]), were localized in the last hundreds of kilobases, confirming that DNA rearrangements mostly affected the terminal regions of the linear chromosome.

### Nucleotide sequence accession number.

The genome sequence has been deposited at GenBank under the accession no. CP012949. Strain DSM 40697 is available from Deutsche Sammlung von Mikroorganismen und Zellkulturen (DSMZ).
